# Actively Tunable “Single Peak/Broadband” Absorbent, Highly Sensitive Terahertz Smart Device Based on VO_2_

**DOI:** 10.3390/mi15020208

**Published:** 2024-01-30

**Authors:** Baodian Fan, Hao Tang, Pinghui Wu, Yu Qiu, Linqin Jiang, Lingyan Lin, Jianzhi Su, Bomeng Zhou, Miao Pan

**Affiliations:** 1 Key Laboratory of Green Perovskites Application of Fujian Provincial Universities, Fujian Jiangxia University, Fuzhou 350108, China; fanbd@fjjxu.edu.cn (B.F.); yuqiu@fjjxu.edu.cn (Y.Q.); linqinjiang@fjjxu.edu.cn (L.J.); lingyanlin@fjjxu.edu.cn (L.L.); 2 Key Laboratory of Information Functional Material for Fujian Higher Education, College of Physics and Information Engineering, Quanzhou Normal University, Quanzhou 362000, China; 18350115109@139.com (H.T.); phwu@zju.edu.cn (P.W.); sujianzhi@126.com (J.S.); simonqz@foxmail.com (B.Z.)

**Keywords:** terahertz, heat regulation, vanadium dioxide, metamaterial, local surface plasmon resonance

## Abstract

In recent years, the development of terahertz (THz) technology has attracted significant attention. Various tunable devices for THz waves (0.1 THz–10 THz) have been proposed, including devices that modulate the amplitude, polarization, phase, and absorption. Traditional metal materials are often faced with the problem of non-adjustment, so the designed terahertz devices play a single role and do not have multiple uses, which greatly limits their development. As an excellent phase change material, VO_2_’s properties can be transformed by external temperature stimulation, which provides new inspiration for the development of terahertz devices. To address these issues, this study innovatively combines metamaterials with phase change materials, leveraging their design flexibility and temperature-induced phase transition characteristics. We have designed a THz intelligent absorber that not only enables flexible switching between multiple functionalities but also achieves precise performance tuning through temperature stimulation. Furthermore, we have taken into consideration factors such as the polarization mode, environmental temperature, structural parameters, and incident angle, ensuring the device’s process tolerance and environmental adaptability. Additionally, by exploiting the principle of localized surface plasmon resonance (LSPR) accompanied by local field enhancement, we have monitored and analyzed the resonant process through electric field characterization. In summary, the innovative approach and superior performance of this structure provide broader insights and methods for THz device design, contributing to its theoretical research value. Moreover, the proposed absorber holds potential for practical applications in electromagnetic invisibility, shielding, modulation, and detection scenarios.

## 1. Introduction

In recent years, the development of terahertz technology has attracted widespread attention [1,2,3] due to its significant potential in areas such as communication, imaging, and sensing [4,5]. Various tunable devices for terahertz waves (0.1 THz–10 THz) have been proposed, including devices that modulate the amplitude, polarization state, phase, and absorption [6,7,8]. However, because most natural materials maintain a stable state in the natural environment for a long time, they do not have any response to terahertz waves, and a few materials such as “gold” can achieve solid absorption of terahertz waves through a resonator, but they are not tunable. The emergence of artificially designed metamaterials and phase change materials breaks this limitation, and they not only have a high absorption but also a good tunability, such as metamaterial graphene, which can achieve dynamic tunability by the application of an external bias voltage, so that terahertz devices can be rapidly developed [9,10,11].

Metamaterials, artificially designed composite materials with unit structures tailored to exhibit extraordinary physical properties not found in natural materials, have been extensively studied since the concept was introduced by Soviet physicist Victor Veselago in 1968 [12,13,14]. Among the various applications of metamaterials, electromagnetic metamaterials have been widely employed, offering unique advantages in electromagnetic wave manipulation, electromagnetic invisibility, communication, the design of terahertz devices, and other fields [15,16,17,18]. Some metamaterials regulate terahertz devices by changing the Fermi level, such as two-dimensional materials like graphene and Dirac semi-metals, by applying an external bias voltage and chemical doping, respectively [19,20,21,22]. However, some metamaterials’ internal structures change through light, heat, and electricity to achieve changes in material properties, thus realizing the regulation of terahertz devices [23,24]. VO_2_ is such a material; its internal structure can be changed by external temperature changes, and it shows different properties (an insulating state and a metallic state) at low and high temperatures. These properties can change the structural composition of terahertz devices, and the structure determines the properties, so reasonable design and scientific research can realize the regulation of terahertz devices [25,26,27,28].

Since the first terahertz metamaterial absorber was designed by N. I. Landy in 2008, the investigation of terahertz metamaterial absorbers has become a research hotspot [29,30,31]. In this paper, we propose a phase-change-material-based metasurface terahertz absorber. By controlling the temperature, the absorber can freely switch between wideband absorption and narrowband absorption. It consists of a simple sandwich structure, including a bottom reflective layer, an intermediate dielectric layer, and a patterned resonant layer (absorption layer) on the surface. The design is supported by an electromagnetic, effective medium and VO_2_ phase transitions, and is simulated using the finite element method. During the design process, by controlling the patterns (boundary states), we achieve perfect absorption in the terahertz range based on the principle of localized surface plasmon resonance. The broadband absorption is attributed to the coupling between the ring structure and the electromagnetic modes of the metallic VO_2_ square disk structure, while narrowband absorption is achieved via the disruption of the coupling due to the phase transition of VO_2_ from the metallic state to the insulating state and the matching of the ring structure with the external terahertz wave resonance frequency.

## 2. Structure and Design

**Figure 1:**
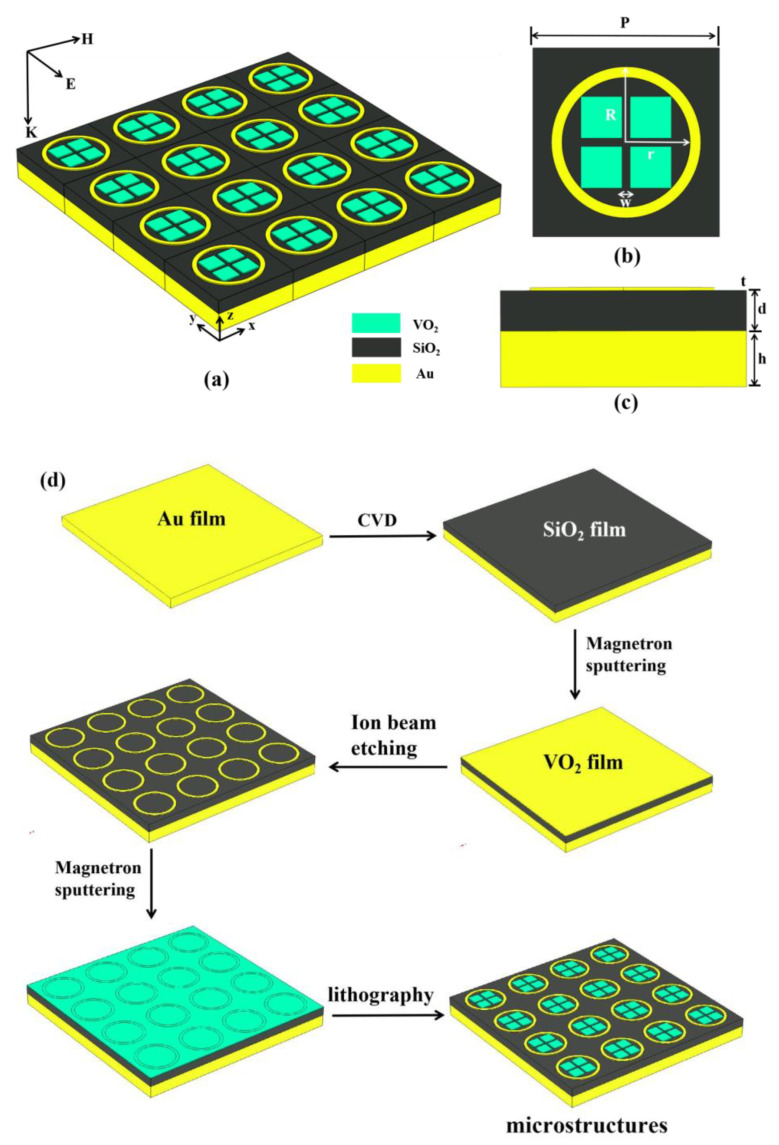
(**a**) Structure array diagram of the TIA; (**b**) xy plane diagram of periodic unit; (**c**) periodic unit yz plane diagram; (**d**) TIA device preparation process flow chart.

The proposed terahertz intelligent absorber (TIA) structure is illustrated in Figure 1. The unit structure with a side length of *P* is periodically repeated in the xy plane. Using COMSOL(6.1) simulation software, the arrangement of this infinite array can be achieved in simulations by setting periodic boundary conditions in the x and y directions of one unit cell. In addition, an open boundary condition is set in the z direction, allowing the electromagnetic wave to incident in the negative direction of the z axis. The device consists of three layers, including a bottom layer of gold thin film, an intermediate layer of dielectric silicon dioxide, and a top layer composed of a combination of metal and VO_2_. The relative permittivity of nondestructive silica is 2.13. The top layer consists of a gold ring and four VO_2_ square blocks connected to it, all having a uniform height of *t*. The inner and outer radii of the ring are denoted as *r* and *R*, respectively, while the channel width between the blocks is represented as *w*.

The Drude model allows characterization of the dielectric parameters of vanadium dioxide in the terahertz band, as shown in Equation (1) [32,33,34]:

(1) $${\varepsilon (\omega )}_{VO2}=\varepsilon_{\infty }-\frac{\omega_{P}^{2}}{\omega (\omega +i\gamma )}$$

(2) $$\omega_{P}^{2}=\frac{\sigma }{\sigma_{0}}\omega_{P0}^{2}$$

Here, $\varepsilon_{\infty }$ is the dielectric parameter at a high frequency, which has a value of 12, and $\omega_{P}$ is the plasma frequency related to conductivity, as illustrated in Equation (2). The initial value of the plasma frequency is $\omega_{P0}\;$= 1.45 $\times \;10^{15}\;s^{-1}$, $\sigma_{0}$ = 3 $\times \;10^{5}\;Sm^{-1}$, and the collision frequency $\gamma =5.75\;\times \;10^{13}\;s^{-1}$. The conductivity σ will vary with the phase change of the vanadium dioxide film. From the relationship between the dielectric constant and the conductivity of the material, we are able to obtain the relational equation for the conductivity of vanadium dioxide films at various temperatures in the phase transition process, as shown in Equation (3) [35,36].

(3) $$\sigma =-i\varepsilon_{0}\omega (\varepsilon_{c}-1)$$

where σ is the conductivity of the compositesystem, $\varepsilon_{0}$ is the dielectric constant of the vacuum, and ω is the angular frequency. The dielectric coefficient of the composite system $\varepsilon_{c}$ is a temperature-dependent function.

When the VO_2_ disk is in the metallic state (*σ* = 200,000 S/m, *T* = 345 K), the VO_2_ blocks, together with the gold ring, form the resonator of the absorption layer. However, when VO_2_ is in the insulating state (*σ* = 100 S/m, *T* = 312 K), the absorption layer is solely composed of the metal ring, while the four VO_2_ blocks are treated as the dielectric layer. The structure is simulated using finite-element-method-based simulation software (COMSOL); the surrounding environment is set to normal, the electromagnetic wave is set to normal incidence, and the simulation range is 7–11 THz. And the end of optimization, structural data were obtained as follows: *P* = 35 μm, *h* = 8 μm, *d* = 5.75 μm, *t* = 0.1 μm, *r* = 12 μm, *R* = 13.5 μm, and *w* = 1.5 μm.

The fabrication process of the TIA device is illustrated in Figure 1d. Following a bottom-up approach, an 8 μm-thick gold film was sputtered onto a prepared substrate, followed by the growth of a 5.75 μm-thick SiO_2_ dielectric layer using chemical vapor deposition (CVD). Subsequently, another layer of gold film with a thickness of 0.1 μm was sputtered, and the desired metal ring structure was obtained through processes such as photolithography. Finally, the vanadium dioxide film was sputtered on the surface of the dielectric layer using the magnetron sputtering method, and the array structure of the TIA was obtained by repeating the lithography process [37,38]. In this way, we present the process of preparing the proposed absorber, showing its feasibility in theory, but its specific experimental data need to be measured in practice. Since this is not the focus of our paper, it is not described in detail in this paper.

## 3. Simulation Results and Discussion

**Figure 2:**
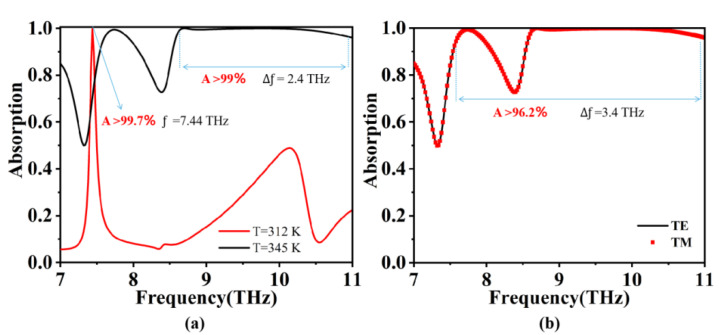
Schematic diagram of the spectral curve of the TIA. (**a**) Absorption curves of vanadium dioxide cube in the metallic state (*T* = 345 K) and the insulating state (*T* = 312 K); (**b**) absorption spectra of TIA devices in TE and TM modes.

Using numerical simulations, we calculated the *S*11 parameter, and we used the *S*11 parameter to calculate the absorption rate. The absorption rate *A* = 1 − *R* − *T*, where *T* represents transmission. As a result of the bottom metal plate, an electromagnetic wave cannot be transmitted, so *T* = 0 and *R* represents a refraction, where *R* = |*S*11|^2^, and so *A* = 1 − |*S*11|^2^ [39,40,41]. We performed simulations to calculate the absorption properties of the absorber under different polarization modes (TE and TM) and temperatures (where the vanadium dioxide square undergoes a phase transition), as illustrated in Figure 2. The results revealed that the absorber device exhibits an average absorption rate exceeding 96.2% across an ultra-wideband range of 7.5–11 THz. Notably, this device demonstrates a remarkable absorption bandwidth of 3.5 THz and possesses polarization-insensitive characteristics [42,43]. Moreover, by leveraging temperature control, the absorber can flexibly switch between narrowband and ultra-wideband absorption modes. Specifically, when the vanadium dioxide disk is in the metallic state (*T* = 345 K), we propose the absorber achieves an average absorption rate exceeding 99% within the ultra-wideband range of 8.6–11 THz. In contrast, when the carbon dioxide disk is in the state of insulation (*T* = 312 K), the absorber forms a narrowband absorption peak with a high quality factor. Remarkably, at an electromagnetic wave frequency of *f* = 7.44 THz, the absorptivity surpasses 99.7%, with a quality factor of 74.4.

**Figure 3:**
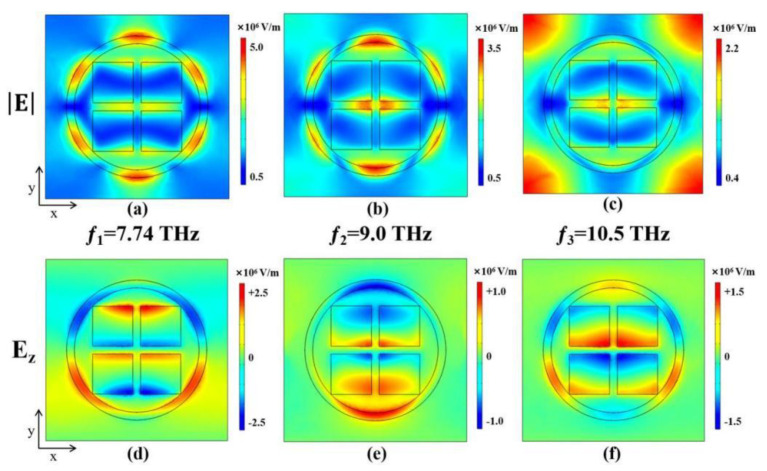
(**a**–**c**) and (**d**–**f)** show the electric field distribution and the electric field component *E_z_* distribution on the surface of the absorber at frequencies *f* = 7.74 THz, *f* = 9.0 THz, and *f* = 10.5 THz, respectively.

To investigate the intrinsic mechanism behind the ultra-wideband absorption exhibited by the absorber when vanadium dioxide is in its metallic state, we conducted simulations and plotted the surface electric field and its components at different frequencies, as illustrated in Figure 3. The observations revealed a distinct distribution of the internal electric field within the absorber on both the gold ring and the vanadium dioxide square at lower and mid-range frequencies. However, as the frequency transitioned from the mid-range to high frequencies, the field strength inside the absorber shifted towards the center, with a more concentrated and stronger electric field on the vanadium dioxide disk compared to the gold ring. This indicates that the resonant response at high frequencies is primarily contributed by the vanadium dioxide square. In fact, the combination of the gold ring and the four vanadium dioxide squares forms a resonant structure, which is responsible for the broadband absorption. When the absorber interacts with external electromagnetic waves, the resonant structure resonates with specific frequencies of the external electromagnetic waves. This resonance results in the generation of absorption peaks at certain frequencies. The appropriate design of the resonant structure enables the absorber to possess multiple resonance frequencies, each leading to a strong absorption. Furthermore, under the excitation of external terahertz waves, the interaction and coupling between the excited electromagnetic modes on the TIA structure cause the resonance frequencies to be closely spaced, resulting in overlapping and coinciding absorption peaks, ultimately achieving broadband absorption [44]. Thus, it can be concluded that the combination of the circular ring and the square disk constitutes a successful resonant structure.

**Figure 4:**
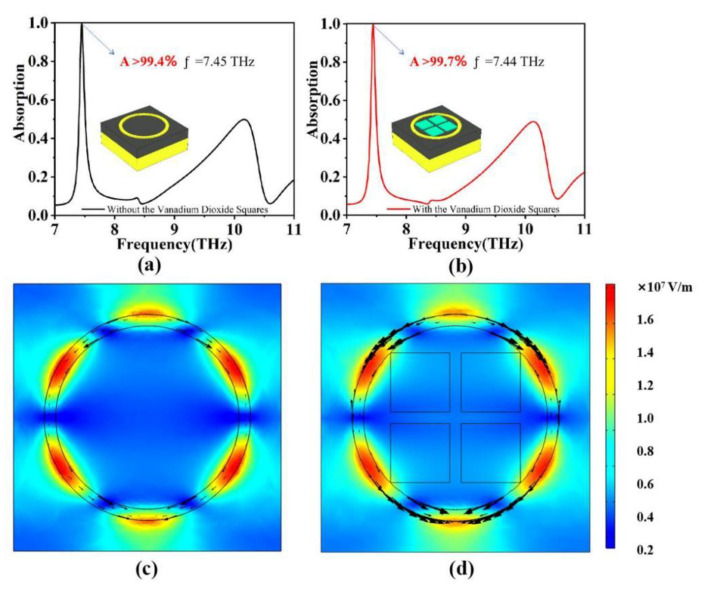
(**a**,**b**) are the absorption spectra of the two structures at *T* = 312 K, respectively. (**c**,**d**) are the surface electric field distributions of the two structures |*E*| when *f* = 7.44 THz.

Subsequently, the intrinsic mechanism behind the narrowband absorption of the absorber in the insulating state of vanadium dioxide was investigated. Figure 4a,b depict the absorption characteristics of the absorber with and without the vanadium dioxide disk, respectively (with the vanadium dioxide disk in the insulating state). It is evident that both structures exhibit a resonance frequency at *f* = 7.45 THz. The first structure achieves a peak absorption rate of >99.4%. The absorber consists solely of a gold ring, which acts as a resonator. Upon interaction between the terahertz wave at *f* = 7.44 THz and the gold ring structure, a resonance effect manifests, resulting in localized field enhancements, as illustrated in Figure 4c. This resonance effectively establishes a dipole oscillation mode, tightly confining the energy of the incident terahertz wave in the near field, substantially depleting the incident wave’s energy and thereby generating a resonance absorption peak at *f* = 7.44 THz.

The second structure achieves a peak absorption rate of >99.7%. In this case, as the vanadium dioxide disk is in the insulating state, its role is equivalent to that of a dielectric layer. The structure of the absorber is analogous to the scenario when only the gold ring is present. The absorption peak can be explained in the same way, but the overall absorber performance in the second structure surpasses that of the first structure. By comparing the absorbers in both structures presented in Figure 4, it is evident that the surface current density and electric field intensity in the second structure surpass those in the first structure. This enhancement is attributed to the presence of the vanadium dioxide disk, which amplifies the resonance effect of the gold ring to a certain extent [45]. According to the classical three-layer structure of the absorber, the electromagnetic coupling caused by the reverse current on the upper and lower surfaces will limit the ability of the electromagnetic wave to reach the middle dielectric layer, so that at low temperatures, VO_2_ exhibits an insulating state and works with the middle dielectric layer so that more energy is limited and a higher absorption peak is generated.

**Figure 5:**
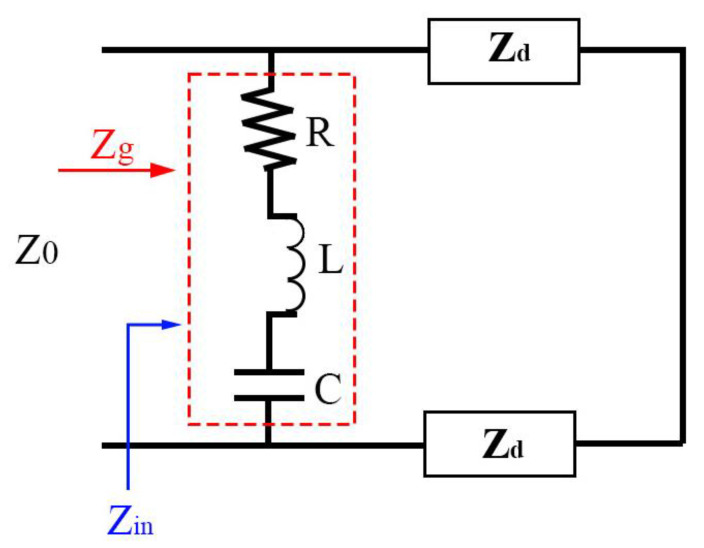
Equivalent resonant circuit of the structural unit.

In addition, we introduce the equivalent circuit diagram to explain the two absorption modes, as shown in Figure 5. When the external electromagnetic wave is incident, the induced current of the device is excited by the upper and lower metal layers. Treating the underlying metal layer as a wire, the equivalent impedance of the intermediate dielectric layer is regarded as *Z_d_*, which is related to its dielectric constant. The equivalent resistance of the top microstructure is regarded as *Z_g_*, and *Z_g_* is expressed as *Z_g_* = *R* + *jωL* − *j*/*ωc*, *ω* = 2πf. This indicates the frequency, and the value of *Z_g_* is affected by the structure and size of the microstructure. Because the external free impedance *Z*_0_ is 377 Ω, when the overall impedance of the device is equal to the external free impedance, the impedance matching condition of the absorber can be achieved, and the maximum absorption benefit is formed. We can adjust the size of *Z_g_* by changing the formation of the top microstructure and the properties of the internal structure, so that the overall absorption system can meet the impedance matching conditions. According to transmission line theory, the transmission matrix of the top VO_2_ microstructure and the middle dielectric layer can be expressed as [46,47]:

(4) $$\left(\begin{array}{cc} 1 & 0\\ \frac{1}{R+j\omega L-j/\omega C} & 1 \end{array}\right)$$

(5) $$\left(\begin{array}{cc} \cos h\gamma h & -Z\sin h\gamma h\\ -\frac{1}{Z}\sin h\gamma h & \cos h\gamma h \end{array}\right)$$

where *Z* represents the normalized characteristic impedance, γ represents the transmission coefficient of the material, which is related to the free space permeability and its own dielectric constant, and *h* represents the thickness of the material. When the impedance matching condition is reached, the relative impedance will be 1. Finally, we can see from simple observations that the impedance value of the top VO_2_ layer can be adjusted between 10 to the fourth power and 10 the first power. Now that our structure is fixed, the process is regulated by changing the internal structural properties of VO_2_. At both low and high temperatures, it can be combined with *Z_d_* to achieve impedance matching conditions, resulting in an electromagnetic response and perfect absorption.

**Figure 6:**
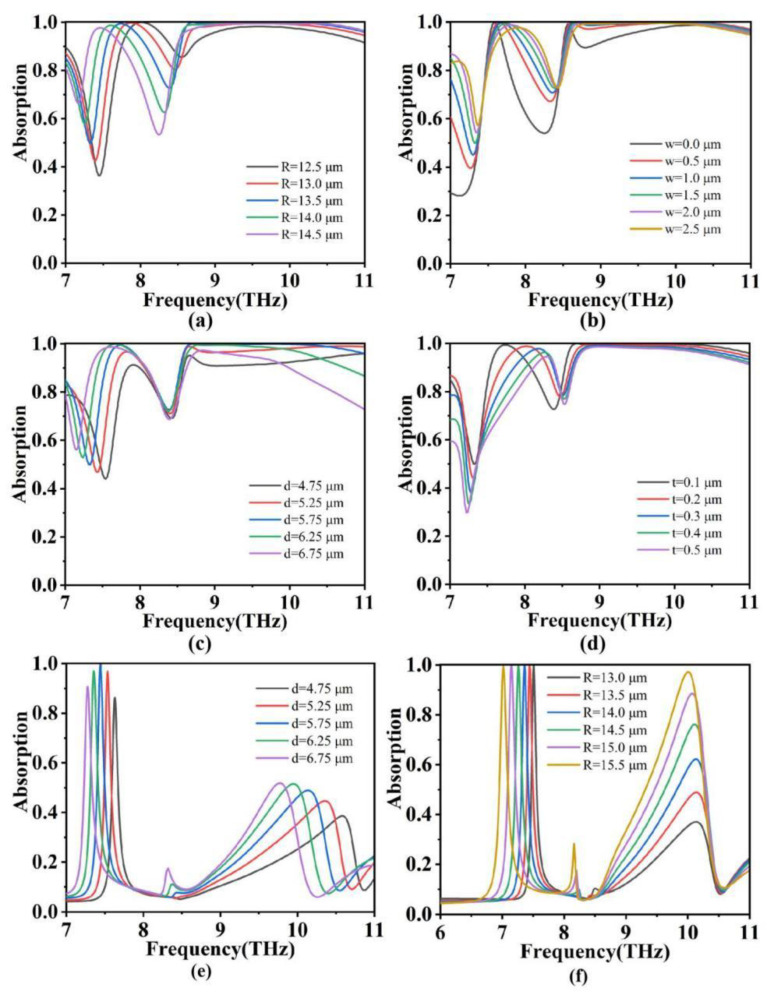
(**a**–**d**) are the scanning graphs of four main parameters affecting the broadband absorption performance of TIA devices at *T* = 345 K. (**e**,**f**) are the scans of two main parameters affecting the narrowband absorption performance of TIA devices at *T* = 312 K.

In the preceding discussion, we delved into the absorption efficiency and underlying mechanisms of the absorber under ideal conditions. However, in practical applications, there often exists a discrepancy between the structural parameters and theoretical models, which can potentially impact the absorption efficiency of the absorber [48,49,50]. To better adapt the absorber to real-world production, we have conducted separate investigations of the influence of structural parameters on the absorption efficiency at temperatures of *T* = 345 K and *T* = 312 K. At *T* = 345 K, based on an analysis of Figure 6a,b,d, it can be observed that the absorption efficiency at high frequencies remains largely unaffected by variations in the outer ring radius ‘*R*’ and the gap distance ‘*w*’ between the metal outer ring and VO_2_, as well as their thickness ‘*t*’. This behavior can be attributed to the dominant contribution of the VO_2_ square in the high-frequency resonance response. However, in the case in Figure 6c, the high-frequency absorption efficiency exhibits an initial increase followed by a decrease with an increasing gap distance ‘*d*’. This can be attributed to the enhanced energy confinement with the introduction of an additional intermediate dielectric layer, but when the thickness reaches a certain threshold, the coupling efficiency between the dielectric layer and external specific electromagnetic waves starts to affect the resonance of the top structure. Furthermore, it is also observed that increasing the outer ring radius ‘*R*’ and the gap distance ‘*d*’ leads to a redshift in the absorption spectrum at mid-to-low frequencies, while increasing the VO_2_ thickness *t* and the gap distance ‘*w*’ results in a blueshift of the absorption peak at mid-to-low frequencies. These findings provide additional avenues for optimizing the structure of our absorber. At *T* = 312 K, it is evident from Figure 6e,f that with an increasing gap distance *d* and outer ring radius ‘*R*’, a noticeable redshift is observed in the mid-to-low frequency absorption spectrum, while the absorption peak at high frequencies also exhibits an increase. At this temperature, the vanadium dioxide (VO_2_) disc is in an insulating state, acting as a dielectric layer. The absorption efficiency of the absorber primarily depends on the top gold ring, and the enlargement of the outer radius enhances the localized surface plasmon resonance, leading to a rapid increase in the absorption peak at high frequencies [51,52]. Furthermore, the increase in the thickness of the dielectric layer can still be explained using classical magnetic resonance theory.

**Figure 7:**
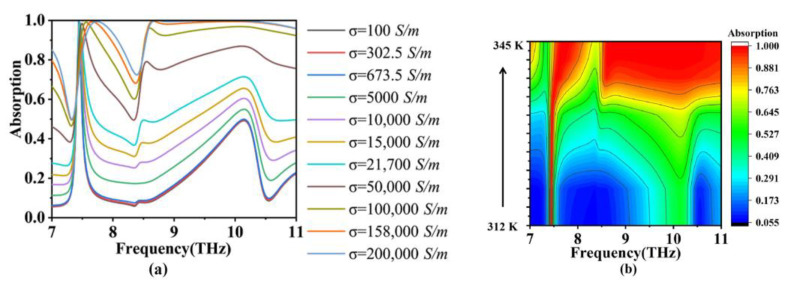
The absorber is affected by different temperatures. (**a**) Schematic drawings of absorption curves at different temperatures; (**b**) the absorption curve corresponding to the contour.

In addition to considering the geometric parameters, we also studied the absorption performance of the absorber at different temperatures, as shown in Figure 7. It can be observed from the figure that when the conductivity ‘*σ*’ of the vanadium dioxide (VO_2_) square varies in the range of 50,000–200,000 S/m, the absorber achieves broadband absorption in the range of 8.5–11 THz. Within this range of conductivity, the VO_2_ square behaves as a metal. The absorber exhibits a traditional metal–dielectric–metal structure. By carefully designing the structural parameters, the equivalent impedance of the TIA matches that of free space, resulting in almost zero reflection of the incident terahertz waves. As a result, the energy of the incident waves can only be continuously dissipated within the TIA device. The absorption rate of electromagnetic waves is close to 100% in a wide frequency range [53,54,55]. On the other hand, when the conductivity ‘*σ*’ of the VO_2_ square varies in the range of 100–10,000 S/m, the absorber achieves narrowband absorption near 7.44 THz. Within this conductivity range, the VO_2_ disc acts as an insulator, playing a role equivalent to a dielectric layer. The insulating state of the VO_2_ disc disrupts the impedance matching condition between the TIA device and free space, resulting in the strong reflection of incident terahertz waves and a decrease in the overall absorption performance of the TIA. However, significant localized electric resonances still exist, forming a narrow absorption peak. Therefore, by controlling the temperature, the absorber can flexibly switch between narrowband and ultra-broadband absorption.

At the same time, we can also observe from Figure 7 that the absorption rate of the TIA devices changes gradually in the process of conductivity changes. When the conductivity is 200,000, the broadband absorption displayed by the TIA will gradually transition to a perfect absorption at 7.44 THz with a gradual decline in the conductivity. In fact, since there is a 6 K temperature difference between the heating and cooling of VO_2_, this transition is also very stable. This perfect regulatory capability enables the absorption rate from 0.1 to 1 to be modulated in the wideband range, enabling our proposed TIA to detect arbitrary signals between the fixed frequency and the wideband range.

**Figure 8:**
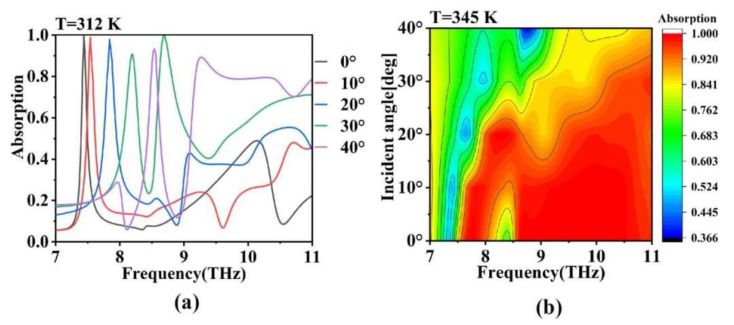
Absorption spectra of the absorber at (**a**) *T* = 312 K and (**b**) *T* = 345 K, where the incidence angle of the light source increases from 0° to 40°, and the corresponding contour diagram of the absorption spectra.

Considering the practical application environment, absorbers are often exposed to oblique incident waves rather than solely vertical plane waves. Therefore, it is necessary to investigate the absorber’s performance under inclined incidence. Based on this, we studied the variations in the absorption spectra of the absorber at temperatures of *T* = 312 K and *T* = 345 K for incident angles ranging from 0° to 40°, as shown in Figure 8a,b. It can be observed that at lower temperatures, as the incident angle increases, the narrowband absorption peak exhibits a blueshift, while the overall absorption performance remains relatively stable. This demonstrates the high sensitivity of the absorber in the field of sensing. At higher temperatures (*T* = 345 K), the TIA maintains a good performance within the range of 0° to 20°, but the absorption bandwidth and intensity gradually decrease with an increasing incident angle, indicating that the designed TIA device is angle-sensitive and requires attention in practical applications [56,57,58]. The rational use of this process can realize the accurate inspection of the signal during the heating process and large-scale detection during the cooling process. However, compared to previous reports, the designed TIA exhibits significant advantages and improvements, as shown in Table 1 [59,60,61,62]. Not only does the absorber have a significantly broadened bandwidth, but it also has an enhanced absorption performance. Additionally, the absorber offers a wide modulation range and can flexibly switch between ultra-broadband absorption and narrowband absorption. These characteristics greatly increase its potential applications in areas such as terahertz sensing and stealth technology.

## 4. Conclusions

Based on a (metal + VO_2_)–dielectric–metal structure, we have designed a THz intelligent absorber that combines ultra-wideband absorption and narrowband absorption. By changing the temperature, the absorber can be arbitrarily adjusted between ultra-wideband and narrowband absorption. As a narrowband absorber, it achieves an absorption peak with a rate greater than 99.9% at *f* = 7.44 THz and a quality factor of 74.4. When used as a broadband absorber, it also achieves perfect absorption close to 100% in the range of 8.5–11 THz, exhibiting polarization insensitivity. To investigate the mechanism of broadband absorption, we have depicted the electric field distribution of the absorber at different frequencies. According to the LSPR principle, the reasons for formation of the two absorption modes are explained. It is found that the metallic vanadium dioxide disk and gold ring form a well-defined resonator. We simulated the effect of the structural parameters of the absorber on the absorption performance and observed that the absorber has tolerance in the manufacturing process. The results also provide more direction for the adjustability of the absorber. By modifying the absorber’s layer structure, we have analyzed the reasons for the narrowband absorption peak. Furthermore, we have explored the performance of the absorber at different incident angles and discovered its high sensitivity to angle variations. In summary, the proposed THz smart absorber, referred to as a TIA, exhibits dynamic tunability, contactless adjustment, multifunctionality, polarization independence, and low-angle insensitivity, offering superior absorption bandwidth and enhanced control capabilities compared to previous similar absorbers. Therefore, it holds great potential for applications in the THz frequency range, such as sensing and stealth technologies.

## Figures and Tables

**Table 1:** Some coordinated terahertz absorbers in recent years.

References	Coordinated Type	Absorption Range	Absorption
[59]	Dual broadband coordination	0.76–0.86 THz 1.12–1.25 THz	More than 90%
[60]	Two-band absorption	5.5 THz, 8.5 THz	More than 95%
[61]	Broadband coordination	8.62–10 THz	More than 95%
[62]	Three-band absorption	3.56 THz, 10.38 THz, 12.96 THz	More than 95%
proposed	Ultra wideband and single-band coordination	7.5–11 THz	More than 95%

## Data Availability

Publicly available datasets were analyzed in this study. These data can be found here: [https://www.lumerical.com/ (accessed on 1 January 2020)].

## References

[1] Li W.X., Zhao W.C., Cheng S.B., Yang W.X., Yi Z., Li G.F., Zeng L.C., Li H.L., Wu P.H., Cai S.S. (2023). Terahertz Selective Active Electromagnetic Absorption Film Based on Single-layer Graphene. Surf. Interfaces.

[2] Chernomyrdin N.V., Musina G.R., Nikitin P.V., Dolganova I.N., Kucheryavenko A.S., Alekseeva A.I., Wang Y., Xu D., Shi Q., Tuchin V.V. (2023). Terahertz technology in intraoperative neurodiagnostics: A review. Opto-Electron. Adv..

[3] Zheng Z., Luo Y., Yang H., Yi Z., Zhang J., Song Q., Yang W., Liu C., Wu X., Wu P. (2022). Thermal tuning of terahertz metamaterial properties based on phase change material vanadium dioxide. Phys. Chem. Chem. Phys..

[4] Kürner T. (2012). Towards future THz communications systems. Terahertz Sci. Technol..

[5] Li W., Yi Y., Yang H., Cheng S., Yang W.X., Zhang H., Yi Z., Yi Y., Li H. (2023). Active Tunable Terahertz Bandwidth Absorber Based on single layer Graphene. Commun. Theor. Phys..

[6] Li J.T., Wang G.C., Yue Z., Liu J.Y., Li J., Zheng C., Zhang Y., Zhang Y., Yao J. (2022). Dynamic phase assembled terahertz metalens for reversible conversion between linear polarization and arbitrary circular polarization. Opto-Electron. Adv..

[7] Ma J., Tian Y., Cheng J., Cheng S., Tang B., Chen J., Yi Y., Wu P., Yi Z., Sun T. (2023). Active Broadband Absorber Based on Phase-Change Materials Optimized via Evolutionary Algorithm. Coatings.

[8] Zheng R.Y., Liu Y.H., Ling L., Sheng Z.X., Yi Z., Song Q.J., Tang B., Zeng Q.D., Chen J., Sun T.Y. (2024). Ultra wideband tunable terahertz metamaterial absorber based on single-layer graphene strip. Diam. Relat. Mater..

[9] Khonina S.N., Kazanskiy N.L., Khorin P.A., Butt M.A. (2021). Modern Types of Axicons: New Functions and Applications. Sensors.

[10] Cao T., Lian M., Chen X.Y., Mao L.B., Liu K., Jia J., Su Y., Ren H., Zhang S., Xu Y. (2022). Multi-cycle reconfigurable THz extraordinary optical transmission using chalcogenide metamaterials. Opto-Electron. Sci..

[11] Liu T., Liu Y., Ling L., Sheng Z., Yi Z., Zhou Z., Yang Y., Tang B., Zeng Q., Sun T. (2024). Multifunctional terahertz device with active switching between bimodal perfect absorption and plasmon-induced transparency. Mater. Res. Bull..

[12] Ornes S. (2013). Metamaterials. Proc. Natl. Acad. Sci. USA.

[13] Gigli C., Leo G. (2022). All-dielectric χ(2) metasurfaces: Recent progress. Opto-Electron. Adv..

[14] He L., Yi Y., Zhang J., Xu X., Tang B., Li G., Zeng L., Chen J., Sun T., Yi Z. (2024). A four-narrowband terahertz tunable absorber with perfect absorption and high sensitivity. Mater. Res. Bull..

[15] Padilla W.J., Taylor A.J., Highstrete C., Lee M., Averitt R.D. (2006). Dynamical Electric and Magnetic Metamaterial Response at Terahertz Frequencies. Phys. Rev. Lett..

[16] Balci O., Polat E.O., Kakenov N., Kocabas C. (2015). Graphene-enabled electrically switchable radar-absorbing surfaces. Nat. Commun..

[17] Lu W., Yi Z., Zhang J., Xu X., Tang B., Li G., Zeng L., Chen J., Sun T. (2023). A tunable broadband absorber in the terahertz band based on the proportional structure of a single layer of graphene. Diam. Relat. Mater..

[18] Zhang Y.X., Pu M.B., Jin J.J., Lu X.J., Guo Y.H., Cai J., Zhang F., Ha Y., He Q., Xu M. (2022). Crosstalk-free achromatic full Stokes imaging polarimetry metasurface enabled by polarization-dependent phase optimization. Opto-Electron. Adv..

[19] Li A.D., Chen W.J., Wei H., Lu G.W., Alù A., Qiu C.W., Chen L. (2022). Riemann-Encircling Exceptional Points for Efficient Asymmetric Polarization-Locked Devices. Phys. Rev. Lett..

[20] Huang Z., Zheng Y., Li J., Cheng Y., Wang J., Zhou Z.K., Chen L. (2023). High-Resolution Metalens Imaging Polarimetry. Nano Lett..

[21] Li W., Ma J., Zhang H., Cheng S., Yang W., Yi Z., Yang H., Zhang J., Wu X., Wu P. (2023). Tunable broadband absorber based on a layered resonant structure with a Dirac semimetal. Phys. Chem. Chem. Phys..

[22] Fan J.X., Li Z.L., Xue Z.Q., Xing H.Y., Lu D., Xu G., Gu J., Han J., Cong L. (2023). Hybrid bound states in the continuum in terahertz metasurfaces. Opto-Electron. Sci..

[23] Watts C.M., Liu X., Padilla W.J. (2012). Metamaterial Electromagnetic Wave Absorbers. Adv. Mater..

[24] Zhou S., Bi K., Li Q., Mei L., Niu Y., Fu W., Han S., Zhang S., Mu J., Tan L. (2023). Patterned Graphene-Based Metamaterials for Terahertz Wave Absorption. Coatings.

[25] Liang S.R., Xu F., Li W.X., Yang W.X., Cheng S.B., Yang H., Chen J., Yi Z., Jiang P.P. (2023). Tunable smart mid infrared thermal control emitter based on phase change material VO2 thin film. Appl. Therm. Eng..

[26] Kim M.K., Lee D.S., Yang Y.H., Rho J.S. (2021). Switchable diurnal radiative cooling by doped VO_2_. Opto-Electron. Adv..

[27] Ma C.T., Kittiwatanakul S., Sittipongpittaya A., Wang Y., Morshed M.G., Ghosh A.W., Poon S.J. (2023). Phase Change-Induced Magnetic Switching through Metal–Insulator Transition in VO_2_/TbFeCo Films. Nanomaterials.

[28] Lin W., Tang C., Wang F., Zhu Y., Wang Z., Li Y., Wu Q., Lei S., Zhang Y., Hou J. (2023). Building Low-Cost, High-Performance Flexible Photodetector Based on Tetragonal Phase VO_2_ (A) Nanorod Networks. Materials.

[29] Landy N.I., Sajuyigbe S., Mock J.J., Smith D.R., Padilla W.J. (2008). Perfect metamaterial absorber. Phys. Rev. Lett..

[30] Shangguan Q., Zhao Y., Song Z., Wang J., Yang H., Chen J., Liu C., Cheng S., Yang W., Yi Z. (2022). High sensitivity active adjustable graphene absorber for refractive index sensing applications. Diam. Relat. Mater..

[31] Huang Y.J., Xiao T.X., Chen S., Xie Z.W., Zheng J., Zhu J., Su Y., Chen W., Liu K., Tang M. (2023). All-optical controlled-NOT logic gate achieving directional asymmetric transmission based on metasurface doublet. Opto-Electron. Adv..

[32] Cakmak A.O., Colak E., Serebryannikov A.E. (2024). Using Thin Films of Phase-Change Material for Active Tuning of Terahertz Waves Scattering on Dielectric Cylinders. Materials.

[33] Negm A., Bakr M.H., Howlader M.M.R., Ali S.M. (2023). Deep Learning-Based Metasurface Design for Smart Cooling of Spacecraft. Nanomaterials.

[34] Zhu W.L., Yi Y.T., Yi Z., Bian L., Yang H., Zhang J.G., Yu Y., Liu C., Li G.F., Wu X.W. (2023). High confidence plasmonic sensor based on photonic crystal fiber with U-shaped detection channel. Phys. Chem. Chem. Phys..

[35] Li X., Zhang Y., Jiang J., Yao Y., He X. (2023). Full-Space Wavefront Shaping of Broadband Vortex Beam with Switchable Terahertz Metasurface Based on Vanadium Dioxide. Nanomaterials.

[36] Wang D.Y., Zhu W.L., Yi Z., Ma G.L., Gao X., Dai B., Yu Y., Zhou G.R., Wu P.H., Liu C. (2022). Highly sensitive sensing of a magnetic field and temperature based on two open ring channels SPR-PCF. Opt. Express.

[37] Miroshnichenko I.P., Parinov I.A., Chang S.-H., Lin C.-F. (2021). Features and Functionality of the Optical Interference Meter for Measurement of Surface Displacements of Control Objects. Coatings.

[38] Zhang C., Yi Y., Yang H., Yi Z., Chen X., Zhou Z., Yi Y., Li H., Chen J., Liu C. (2022). Wide spectrum solar energy absorption based on germanium plated ZnO nanorod arrays: Energy band regulation, Finite element simulation, Super hydrophilicity, Photothermal conversion. Appl. Mater. Today.

[39] Koschny T., Kafesaki M., Economou E.N., Soukoulis C.M. (2004). Effective medium theory of left-handed materials. Phys. Rev. Lett..

[40] Dittrich S., Spellauge M., Barcikowski S., Huber H.P., Gökce B. (2022). Time resolved studies reveal the origin of the unparalleled high efficiency of one nanosecond laser ablation in liquids. Opto-Electron. Adv..

[41] Shangguan Q., Chen H., Yang H., Liang S., Zhang Y., Cheng S., Yang W., Yi Z., Luo Y., Wu P. (2022). A “belfry-typed” narrow-band tunable perfect absorber based on graphene and the application potential research. Diam. Relat. Mater..

[42] Pacheco-Peña V., Engheta N. (2020). Effective medium concept in temporal metamaterials. Nanophotonics.

[43] Li W.X., Xu F., Cheng S.B., Yang W.X., Liu B., Liu M.S., Yi Z., Tang B., Chen J., Sun T.Y. (2024). Six-band rotationally symmetric tunable absorption film based on AlCuFe quasicrystals. Opt. Laser Technol..

[44] Hendaoui A. (2022). Low Solar Absorptance, High Emittance Performance Thermochromic VO_2_-Based Smart Radiator Device. Nanomaterials.

[45] Ma J., Wu P.H., Li W.X., Liang S.R., Shangguan Q.Y., Cheng S.B., Tian Y.H., Fu J.Q., Zhang L.B. (2023). A five-peaks graphene absorber with multiple adjustable and high sensitivity in the far infrared band. Diam. Relat. Mater..

[46] Wang G., Wu T., Jia Y., Gao Y., Gao Y. (2022). Switchable Terahertz Absorber from Single Broadband to Dual Broadband Based on Graphene and Vanadium Dioxide. Nanomaterials.

[47] Shangguan Q., Chen Z., Yang H., Cheng S., Yang W., Yi Z., Wu X., Wang S., Yi Y., Wu P. (2022). Design of Ultra-Narrow Band Graphene Refractive Index Sensor. Sensors.

[48] Liang S., Xu F., Yang H., Cheng S., Yang W., Yi Z., Song Q., Wu P., Chen J., Tang C. (2023). Ultra long infrared metamaterial absorber with high absorption and broad band based on nano cross surrounding. Opt. Laser Technol..

[49] Javadi F.S., Metselaar H.S.C., Ganesan P. (2020). Performance improvement of solar thermal systems integrated with phase change materials (PCM), a review. Sol. Energy.

[50] Heßler A., Bente I., Wuttig M., Taubner T. (2021). Ultra-Thin Switchable Absorbers Based on Lossy Phase-Change Materials. Adv. Opt. Mater..

[51] Zhang Y., Yi Y., Li W., Liang S., Ma J., Cheng S., Yang W., Yi Y. (2023). High Absorptivity and Ultra-Wideband Solar Absorber Based on Ti-Al2O3 Cross Elliptical Disk Arrays. Coatings.

[52] Buono W.T., Forbes A. (2022). Nonlinear optics with structured light. Opto-Electron. Adv..

[53] Hutter E., Fendler J.H. (2004). Exploitation of Localized Surface Plasmon Resonance. Adv. Mater..

[54] Sepúlveda B., Angelomé P.C., Lechuga L.M., Liz-Marzán L.M. (2009). LSPR-based nanobiosensors. Nano Today.

[55] Wang D.Y., Yi Z., Ma G.L., Dai B., Yang J.B., Zhang J.F., Yu Y., Liu C., Wu X.W., Bian Q. (2022). Two channels photonic crystal fiber based on surface plasmon resonance for magnetic field and temperature dual-parameter sensing. Phys. Chem. Chem. Phys..

[56] Anand V., Han M.L., Maksimovic J., Ng S.H., Katkus T., Klein A., Bambery K., Tobin M.J., Vongsvivut J., Juodkazis S. (2022). Single-shot mid-infrared incoherent holography using Lucy-Richardson-Rosen algorithm. Opto-Electron. Sci..

[57] Li W.X., Liu M.S., Cheng S.B., Zhang H.F., Yang W.X., Yi Z., Zeng Q.D., Tang B., Ahmad S., Sun T.Y. (2024). Polarization independent tunable bandwidth absorber based on single-layer graphene. Diam. Relat. Mater..

[58] Ushanov V.I., Eremeev S.V., Silkin V.M., Chaldyshev V.V. (2024). Unveiling Influence of Dielectric Losses on the Localized Surface Plasmon Resonance in (Al,Ga)As:Sb Metamaterials. Nanomaterials.

[59] Zhao Y., Huang Q., Cai H., Lin X., Lu Y. (2018). A broadband and switchable VO2-based perfect absorber at the THz frequency. Opt. Commun..

[60] Biabanifard S. (2021). A graphene-based dual-band THz absorber design exploiting the impedance-matching concept. J. Comput. Electron..

[61] Huang M., Wei K., Wu P., Xu D., Xu Y. (2021). Terahertz Broadband Absorber Based on a Combined Circular Disc Structure. Micromachines.

[62] Cen C.L., Yi Z., Zhang G.F., Zhang Y.B., Liang C.P., Chen X.F., Tang Y.J., Ye X., Yi Y.G., Wang J. (2019). Theoretical design of a triple-band perfect metamaterial absorber in the THz frequency range. Results Phys..

